# FDG PET/CT for the early prediction of RAI therapy response in patients with metastatic differentiated thyroid carcinoma

**DOI:** 10.1371/journal.pone.0218416

**Published:** 2019-06-25

**Authors:** Seo Young Kang, Ji-In Bang, Keon Wook Kang, Ho-young Lee, June-Key Chung

**Affiliations:** 1 Department of Nuclear Medicine, Seoul National University Hospital, Seoul, Korea; 2 Department of Nuclear Medicine, Ewha Womans University Medical Center, Seoul, Korea; 3 Department of Molecular Medicine and Biopharmaceutical Science, Graduate School of Convergence Science and Technology, Seoul National University, Seoul, Korea; 4 Department of Nuclear Medicine, Seoul National University Bundang Hospital, Seong-Nam, Korea; 5 Department of Nuclear Medicine, National Cancer Center, Goyang, Korea; Wayne State University, UNITED STATES

## Abstract

**Background:**

In some patients with metastatic differentiated thyroid cancer, even if they had substantial of radioactive iodine (RAI) uptake, the RAI therapy response was poor. We investigated the usefulness of FDG PET/CT for the early prediction of RAI therapy response in the patients with metastatic differentiated thyroid cancer (DTC).

**Methods:**

The 54 metastatic DTC patients who underwent both RAI therapy scan and FDG PET/CT at the same period were enrolled in the study. Clinical information and several parameters from RAI therapy scan and FDG PET/CT were investigated. Therapeutic response of RAI was assessed as two categories: response rate (RR) and disease control rate (DCR).

**Results:**

Twenty-two patients (41%) had therapeutic response to RAI therapy, whereas 32 (59%) patients did not. There were no significant differences in age, sex, stage, histology, metastasis site, stimulated Tg or Tg-Ab, therapeutic doses, and even RAI uptake pattern among two groups. However, there was a significant negative correlation between FDG avidity of metastatic lesions and RR (OR = 0.233; p = 0.016). Although the patient group with only RAI uptake showed a significant correlation with RR (OR = 5.833; p = 0.01), the patient group with both RAI and FDG uptake did not show any significant correlation with RR. In the subgroup analysis, uptake grades of RAI or FDG was well correlated with DCR.

**Conclusions:**

The patient group with FDG uptake in metastatic DTC showed poor response to RAI therapy regardless of the degree of RAI uptake. Therefore, FDG PET/CT may help us identify the patients with radioiodine refractory DTC and establish an appropriate treatment strategy in the early period.

## Introduction

The incidence of thyroid cancer has been increasing in many countries including Korea [1]. Metastasis from differentiated thyroid cancer (DTC) occurs in approximately 10% of all patients, and radioactive iodine (RAI) therapy is a well-known first-line therapeutic option [2–4]. Approximately 33%–50% patients with metastasis eventually become refractory to RAI [5, 6] and these patients generally have poor prognosis. The median survival for patients with RAI-refractory DTC and distant metastases is estimated to be 2.5–3.5 years [7, 8]. Recently, tyrosine kinase inhibitor (TKI) medications, such as sorafenib and lenvatinib, have been introduced in these RAI-refractory patients with an expectation of improved prognosis [9, 10]. Therefore, it is important to identify RAI-refractory DTC patients in the early period and establish appropriate treatment strategies from a long-term perspective.

Generally, high uptake of RAI in metastatic carcinoma suggests good therapeutic effect, and several studies have reported that there is a dose–response relationship [11]. However, even if metastatic lesions show substantial RAI uptake, not all the lesions represent therapeutic response. Schlumberger group reported that 295 (68%) of 444 patients with distant metastases showed RAI uptake, and 168 patients (57%) of those patients did not achieve remission [7]. There are several hypothesis to explain this phenomenon, and the main reason for this will be probably that the amount of RAI concentrated in the metastatic thyroid cancer is not sufficient to produce a therapeutic effect. The ability of thyroid cancers to concentrate RAI is dependent on the expression and functional integrity of the sodium-iodide symporter (NIS) [12, 13]. Poorly differentiated thyroid cancers are incapable of concentrating iodide, which renders them refractory to RAI therapy and increases the morbidity and mortality for these patients. Although the degree of cell differentiation of primary thyroid cancer can be confirmed in the surgical tissues, it is practically impossible to confirm the degree of differentiation of all metastatic tissues. Therefore, FDG PET/CT has been suggested as a good way to indirectly determine the degree of differentiation of these cells.

It is well known that FDG uptake depends on the degree of tumor differentiation and proliferation [14–16]. In thyroid cancer, flip-flop phenomenon is representative, which is an inverse relationship between RAI and FDG accumulation in cancer cell [17, 18]. Thus, information from both RAI and FDG scans will help us better assess the differentiation status of metastasis and further predict the treatment effect of RAI.

In this retrospective study, we investigated the roles of FDG PET/CT to predict the response of RAI therapy in the patient with metastatic DTC.

## Patients and methods

### Patients

**Fig 1:**
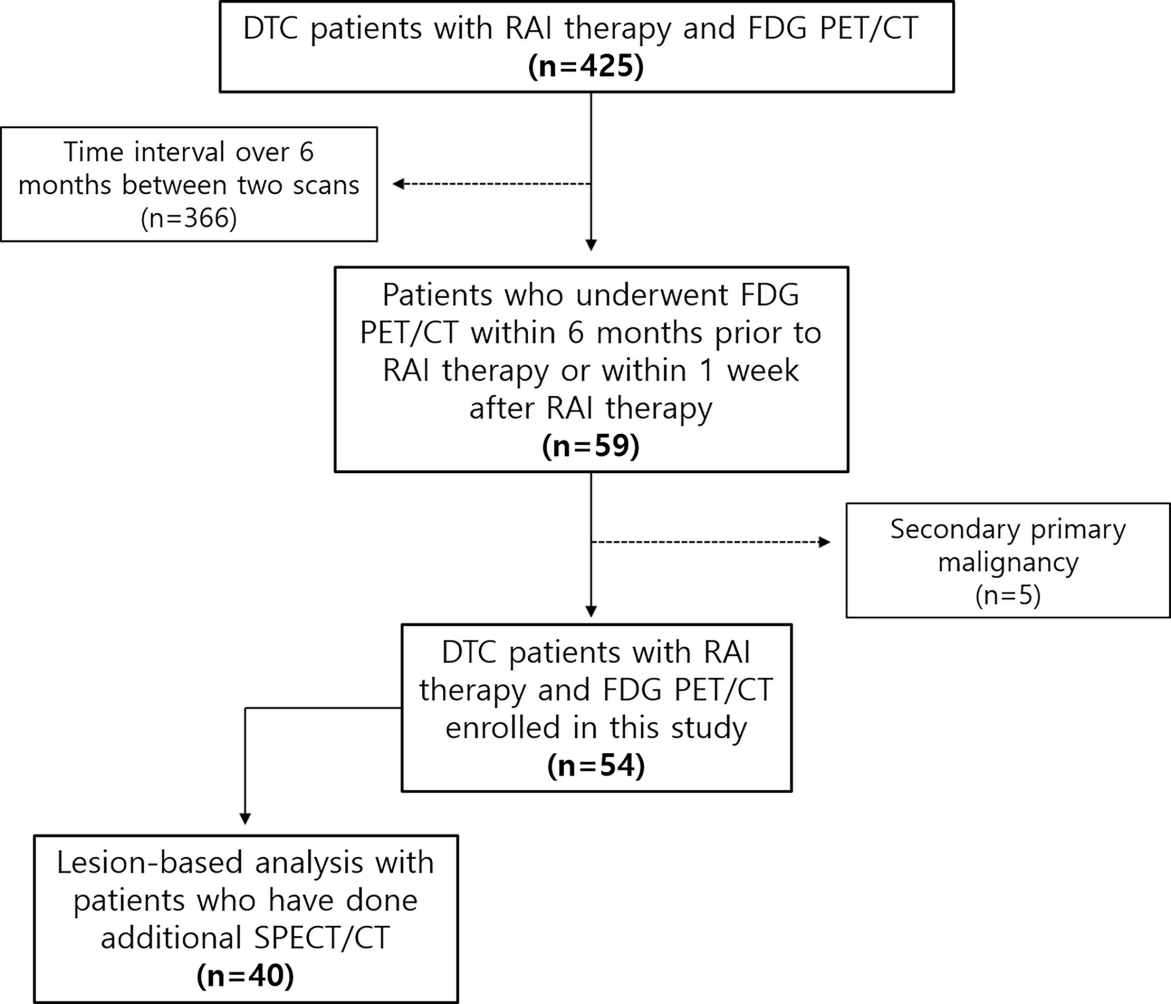
Flow chart of enrollment of study participants.

From March 2007 to December 2017, 425 metastatic DTC patients who underwent both RAI therapy scan and FDG PET/CT in two multicenter were retrospectively reviewed. Among them, 59 patients who underwent FDG PET/CT within 6 months prior to RAI therapy or within 1 week after RAI therapy were selected. Five patients with secondary primary malignancy were excluded in this study. Finally, 54 patients were enrolled in this study (Fig 1). Clinical information including age, sex, histopathology, cancer stage, and serum Tg and Tg-Ab levels of TSH stimulation were investigated. All procedures followed were performed in accordance with the ethical standards of the responsible committee on human experimentation and in agreement with the tenets of the Helsinki Declaration of 1975, as revised in 2013. The study design and exemption of informed consent were approved by the Institutional Review Board of the Seoul National University Hospital (IRB No. 1705-083-855). This study was a retrospective medical record survey, and it was practically impossible to obtain consent from the patient at this time.

### Radioactive iodine therapy

Therapeutic dose of I-131 was administered in conjunction with a strict low-iodine diet using a standard thyroid hormone withdrawal protocol or recombinant human THS (rhTSH) injection. For rhTSH method, the 0.9mg rhTSH was intramuscularly injected twice 24 hours apart and radioiodine administration was performed 24 hours after the last dose. In case of thyroid hormone withdrawal, each patient was required to discontinue levothyroxine supplementation 4 weeks before I-131 therapy and to replace to triiodothyronine for 2 weeks. After then, all forms of thyroid hormone supplementation were prohibited. In the meantime, patients avoided iodine-containing medications and restricted iodine-containing foods for 2 weeks before RAI therapy. In particular, iodide contrast agents were restricted for 2–3 months.

### Post-therapy whole-body scan and analysis

A post-therapy whole-body scan (WBS) was performed 3–5 days after RAI administration. Whole-body images were obtained using a gamma camera equipped with a high-energy, general-purpose collimator (E-CAM; Siemens Medical Systems, Erlangen, Germany). A 10% symmetrical window was centered at 364 KeV, and anterior and posterior images of the whole body were obtained at a table speed of 60 mm per minute. Post-therapy whole-body images were visually interpreted by consensus between two nuclear medicine physicians.

### SPECT/CT imaging protocol

We used an integrated SPECT/CT scanner (NMCT530; GE Healthcare, Pittsburgh, PA, USA) with two detector heads fitted with high-energy, general-purpose collimators. After acquisition of whole-body planar images, SPECT/CT was conducted if additional anatomical information was needed. The SPECT portion was acquired using the following parameters: energy peak, 364 kV with 10% width; acquisition, continuous rotation mode of 180° for each head; projection, a total of 60 over 360° with a dwell time of 30 s/view (a total of 20 min/scan); reconstruction, Ordered subset expectation maximization algorithm with 8 subset and 5 iterations; pixel size, 2.54 × 2.54 × 2.54 mm; and matrix, 256 × 256.

### Lesion-based analysis

A lesion-based analysis was performed using semi-quantification in 40 patients who underwent regional SPECT/CT as well as post therapy whole-body scan. Two or three representative lesions per patient were selected and total 121 lesions were identified. A spherical volume of interest (VOI) was drawn to count the relative amount of radioactive iodine uptake in the metastatic lesions, and maximal count obtained for each lesion was used for analysis. For calculating the target-to-background ratio (TBR), another spherical VOI of 1.5 cm^3^ was drawn from the thoracic aorta while avoiding the arterial wall to assess the blood pool activity. The TBR of metastatic lesions was calculated as the maximal count of each lesion divided by the mean count of thoracic aorta blood pool. The TBRs of metastatic lesions were graded on a scale of 1–3 as follows: grade 1, more than 2 and less than 10; grade 2, more than 10 and less than 50; grade 3, more than 50.

### ^18^F-FDG PET/CT imaging protocol

The patients underwent FDG PET/CT using dedicated PET/CT scanners (Biograph 40 True-point, Siemens, Knoxville, TN, USA; Discovery VCT, GE Medical Systems, Milwaukee, WI, USA). After fasting for at least 6 h, ^18^F-FDG of 5.18 MBq/kg was administered intravenously, and image acquisition was started at 60 min after the injection. Serum glucose levels were less than 150 mg/dL at the time of FDG administration in all patients. Emission scan was acquired after the CT scans in three-dimensional mode at each bed position. PET images were corrected for attenuation and reconstructed onto a matrix of 128 × 128 using the three-dimensional ordered-subsets expectation maximization algorithm (2 iterations, 21 subsets).

### FDG PET data analysis

FDG PET/CT images were evaluated by quantitative method as well as visual analysis using commercial software (Syngo.via, VA 30, Siemens Healthcare, Erlangen, Germany). For visual analysis, clinically proven metastases were reviewed in the maximum-intensity projection (MIP) images or the trans-axial images. For quantification, spherical VOIs were drawn in the metastatic lesions to measure the maximum standardized uptake value (SUVmax). The SUVmax of metastatic lesions was graded on a scale of 1–3 as follows: grade 1, more than 1 and less than 3; grade 2, more than 3 and less than 7; grade 3, more than 7.

### Assessment of therapy response

Therapeutic response was assessed as two categories: response rate (RR) and disease control rate (DCR). Positive RR was defined as a decrease in the size or disappearance of known metastatic lesions in the follow-up imaging, such as CT or MR. Decreased stimulated Tg or stimulated Tg-Ab was also considered a therapeutic response. DCR has been defined to include stable disease group as well as positive RR group.

### Statistical analysis

A comparison between the groups by RAI therapy response was performed using Student t-test for continuous variables and Chi-square test or Fisher’s exact test for categorical variables. Univariate analysis was done using logistic regression analysis. A *p*-value of less than 0.05 was considered statistically significant. All analysis was performed using SPSS software (version 25.0; IBM SPSS, Somers, NY, USA).

## Results

### Patient’s characteristics

Total 54 patients were enrolled in the present study. Median follow-up time was 6.5 years. The patients were divided as two groups depending on the therapeutic response. Of total 54 patients (mean age = 55 ± 15 years, age range = 12–77 years, M:F = 1:1.7), 22 patients (40.7%) showed therapeutic response on RAI therapy. The average age of the responder was 54 years (mean age = 54 ± 17 years, age range = 18–77 years), and 15 of the patients (68%) were women. The responder group had the following tumor types: 12 (55%) papillary, 7 (32%) follicular, and 3 (9%) poorly differentiated carcinomas. The most common metastatic site was lung in 15 patients, followed by lymph node in 5 patients and bone in 5 patients.

The other 32 patients (59.3%), non-responder group showed no changes or disease progression after RAI therapy. The average age at diagnosis of this group was 55 years (mean age = 55 ± 14 years, age range = 12–74 years), and 19 of the patients (59%) were women. They showed 23 (72%) papillary, 8 (25%) follicular, and 1 (3%) poorly differentiated carcinomas. The most common metastatic site was lung in 16 patients, followed by bone in 11 patients and lymph node in 7 patients.

There were no statistically significant differences in age, sex, histopathology, stage, metastatic sites and even RAI uptake ratio of metastatic lesions between the two groups. However, the ratio of FDG-avid metastatic lesions was significantly higher in the non-responders than in the responders (78% vs. 45%, p = 0.016). The results are summarized in Table 1.

**Table 1:** The patient characteristics according to the RAI therapy response.

Characteristics	Total (n = 54)	RAI response (n = 22)	No response (n = 32)	*P*-value
Age at diagnosis (yr)	55±15 (12–77)	54±17 (18–77)	55±14 (12–74)	0.760
Study population (n)				0.511
Male	20	7 (32%)	13 (41%)	
Female	34	15 (68%)	19 (59%)	
Total	54	22 (41%)	32 (59%)	
Pathology (n)				0.295
Papillary	35	12 (55%)	23 (72%)	
Follicular	15	7 (32%)	8 (25%)	
poorly-differentiated	4	3 (13)	1 (3%)	
Stage (n)				0.503
I	10	6 (27%)	4 (13%)	
II	17	5 (23%)	12 (38%)	
III	1	0	1 (3%)	
IV	26	11 (50%)	15 (47%)	
Distant metastasis (n)				0.258
LN	12	5 (23%)	7 (22%)	
Lung	30	15 (68%)	16 (50%)	
Bone	16	5 (23%)	11 (34%)	
The amount of RAI (mCi)	178±51	175±52	186±46	0.478
Stimulated Tg (ng/ml)	1316±3181	955±2407	2196±4424	0.196
Stimulated Tg-Ab (ng/ml)	162±518	197±603	67±83	0.405
I-131 whole body scan				
Positive	37	17 (77%)	20 (63%)	0.255
Negative	17	5 (23%)	12 (37%)	
FDG PET/CT				0.016*
Positive	35	10 (45%)	25 (78%)	
Negative	19	12 (55%)	7 (22%)	

Data are mean ± standard deviation.

* p <0.05, statistically significant

### Concordance rate between RAI and FDG uptake

The uptake pattern of RAI and FDG varied among patients. In 34 (69%) of 54 patients, post-therapy RAI scan showed positive uptake at metastatic sites. The patients were subdivided into four groups. The proportions of patients showing positive for both RAI and FDG, negative for RAI and positive for FDG, positive for RAI and negative for FDG, and negative for both RAI and FDG were 43%, 24%, 26%, and 7%, respectively. When we analyzed the uptake pattern of RAI and FDG in each lesion, the result was not quite different with patient-based analysis. The proportions of lesions showing positive for both RAI and FDG, negative for RAI and positive for FDG, positive for RAI and negative for FDG, and negative for both RAI and FDG were 39%, 22%, 29%, and 10%, respectively.

### FDG uptake in the therapeutic response

Clinical parameters including age, sex, stage, stimulated Tg and Tg-Ab did not have a significant relation with RR. Besides, even RAI uptake of metastatic lesions could not predict the treatment results after RAI therapy [Odd Ratio (OR) = 2.040; 95% confidence interval (CI) = 0.598–6.961; p = 0.255]. However, there was a significantly negative correlation between FDG avidity of metastatic lesions and RR (OR = 0.233; 95% CI = 0.071–0.764; p = 0.016). Being analyzed according to the concordance rate of both tracers, RAI and FDG, the patient group with only RAI uptake showed a significant correlation with RR (OR = 5.833; 95% CI = 1.524–22.330; p = 0.01). Patients who represent uptakes in both tracers, however, did not show a correlation with RR (OR = 0.467; 95% CI = 0.150–1.450; p = 0.188). The results were summarized in Table 2.

**Table 2:** Significance of the several parameters in the response rate (RR) and disease control rate (DCR).

Variables		Response Rate (RR)			Disease Control Rate (DCR)		
		Odd Ratio	95% CI	*P-value*	Odd Ratio	95% CI	*P-value*
Age at diagnosis (year)	< 55 years	1.909	0.630–5.789	0.253	1.212	0.365–4.023	0.753
	≥ 55 years						
Sex	Male						
	Female	0.682	0.218–2.135	0.511	0.669	0.203–2.206	0.509
Stage	I			0.503			0.599
	II	0.278	0.054–1.432	0.126	0.204	0.021–2.018	0.204
	III	0.000	—	1	0	—	1
	IV	0.489	0.111–2.159	0.345	0.223	0.027–2.319	0.25
Iodine uptake	Positive	2.040	0.598–6.961	0.255	3.222	0.939–11.054	0.063†
	Negative						
FDG uptake	Positive	0.233	0.071–0.764	0.016*	0.317	0.077–1.301	0.111
	Negative						
Concordance	Both positive	0.467	0.150–1.450	0.188	1.349	0.408–4.467	0.624
	Iodine only positive	5.833	1.524–22.330	0.01*	7.8	0.925–65.787	0.059†
	FDG only positive	0.347	0.083–1.450	0.147	0.241	0.065–0.900	0.034*
	Both negative	1.5	0.195–11.536	0.697	0.389	0.050–3.036	0.368

* p <0.05, statistically significant

† 0.05 ≤ p < 0.1, significant trend

### FDG uptake in the disease control

Similarly to that observed with RR, neither the RAI uptake of metastasis nor the clinical factors had a significant correlation with DCR; however, there was a positive tendency between RAI uptake of metastasis and DCR (OR = 3.222; 95% CI = 0.939–11.054; p = 0.063). FDG uptake was also not a significant factor in DCR. When it was analyzed according to concordance rate of both tracers, patients with only RAI uptake did not show a significant correlation with DCR but they still showed a positive tendency (OR = 7.8; 95% CI = 0.925–65.787; p = 0.059). Patients with only FDG uptake show a negative correlation with DCR (OR = 0.241; 95% CI = 0.065–0.900; p = 0.034). Patients who showed uptakes in both tracers did not show any correlation with DCR (Table 2).

### Lesion-based analysis

In lesion-based subgroup analysis, both RAI and FDG uptake did not show any correlation with RR. Uptake grades of both tracers were also no relation with RR. However, RAI uptake showed a significant correlation with DCR (OR = 5.2; 95% CI = 1.980–13.657; p = 0.001), and FDG uptake was also a good negative predictor for DCR (OR = 0.095; 95% CI = 0.021–0.428; p = 0.002). Uptake grades of RAI was well correlated with DCR, although the odds ratios (OR) of grade 3 lesions were lower than those of grade 2 lesions. The degree of FDG uptake was significantly correlated with DCR in the grade 2–3 lesions. FDG grade 1 lesions did not have any correlation with DCR. The results were summarized in Table 3.

**Table 3:** The response rate (RR) and disease control rate (DCR) at lesion-based analysis.

Variables		Response Rate (RR)			Disease Control Rate (DCR)		
		Odd Ratio	95% CI	*P-value*	Odd Ratio	95% CI	*P-value*
Iodine uptake	Positive	N/A	N/A	0.998	5.2	1.980–13.657	0.001*
	Negative						
Iodine uptake grading							0.010*
	grade 1	N/A	N/A	0.998	3.934	1.100–14.076	0.035*
	grade 2	N/A	N/A	0.998	8.553	1.720–42.521	0.009*
	grade 3	N/A	N/A	0.998	4.789	1.354–16.936	0.015*
FDG uptake	Positive	0.729	0.332–1.602	0.432	0.095	0.021–0.428	0.002*
	Negative						
FDG uptake grading				0.726			0.001*
	grade 1	0.86	0.358–2.065	0.735	0.181	0.021–1.573	0.121
	grade 2	0.543	0.170–1.738	0.304	0.025	0.003–0.221	0.001*
	grade 3	0.642	0.196–2.102	0.464	0.051	0.006–0.470	0.009*

* p <0.05, statistically significant

## Discussion

**Fig 2:**
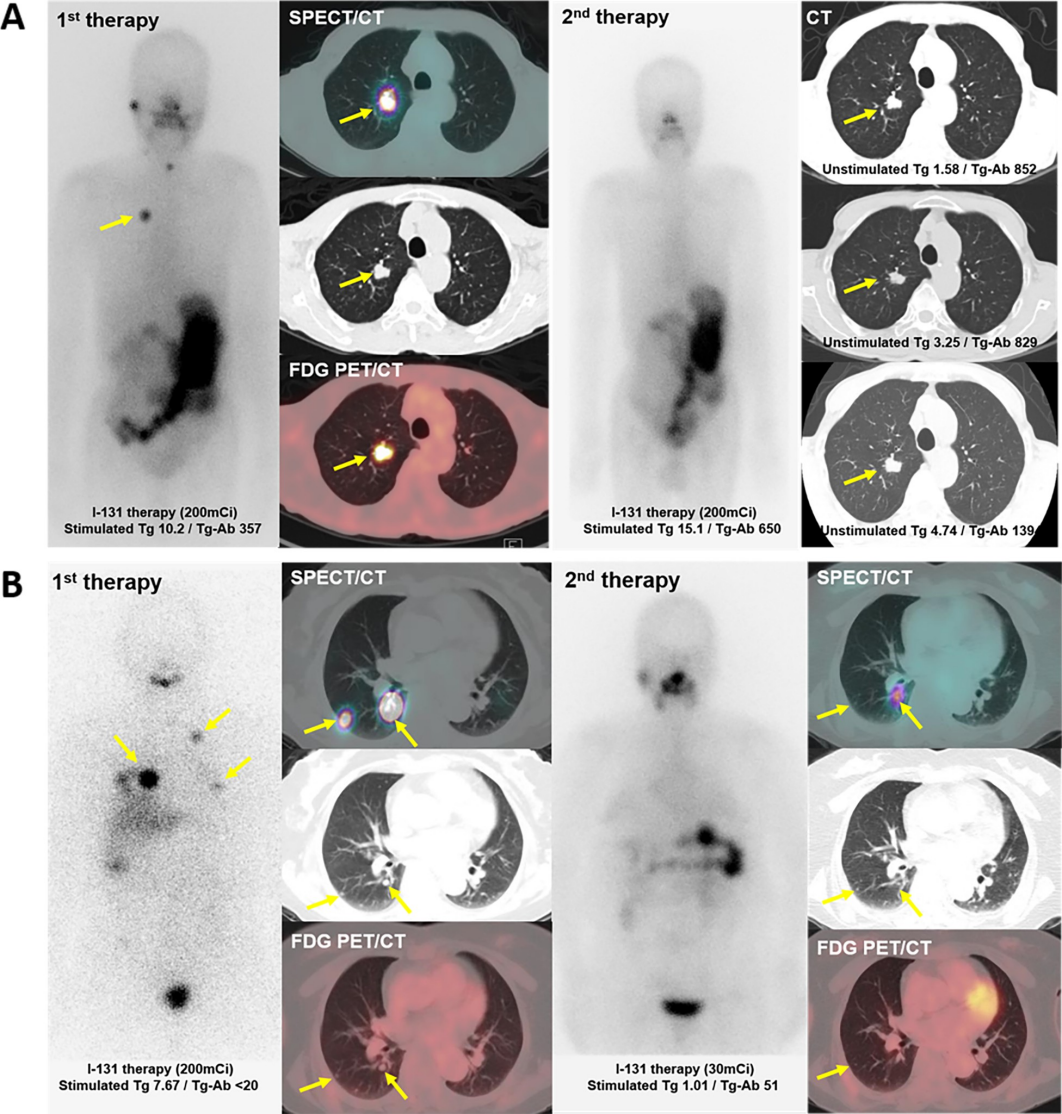
The representative cases showing the predictive role of FDG uptake in RAI therapy. A. The patient who has lung metastasis from papillary thyroid cancer was done RAI therapy after total thyroidectomy. After first therapy, there was quite amount of RAI in the metastatic lesion, expecting therapeutic effect of RAI. However, initial FDG PET scan showed there are also substantial FDG uptake in the metastasis. At the time of 2^nd^ therapy, the RAI uptake was gone and the anatomical size of lung metastasis was not changed during serial follow-up CT scan. Even the unstimulated Tg level as well as stimulated Tg level was subsequently increased. B. The patient who has recurred metastatic lung nodules from papillary thyroid cancer after total thyroidectomy 10 years ago. The metastatic nodules had a significant amount of RAI uptake in the post-therapy scan, but did not show significant uptake in the FDG PET scan performed at the same time. At the time of 2^nd^ therapy, the size of lesions was significantly decreased and stimulated Tg level was also lowered.

We reviewed the uptake patterns of both RAI and FDG in patients with metastatic DTC. RAI uptake of metastasis were not correlated with RR, whereas FDG uptake was negatively correlated with RR. The patient group having only RAI uptake showed a significant correlation with RR, and however, this correlation was not present in the patient group showing uptake of both tracers. These results suggested that FDG uptake of metastasis can be used as a signal of poor therapeutic effect of RAI. That is, FDG-avid metastasis implies that the therapeutic effect of further RAI may be poor even if substantial RAI uptake in the metastatic lesions. The representative cases are in Fig 2.

It is well known that RAI uptake of thyroid carcinoma is related to NIS expression, which changes according to the differentiation of thyroid cancer cells [19, 20]. FDG uptake of thyroid cancer also depends on the degree of differentiation of thyroid cancer cells. Unlike RAI, the lower is the degree of differentiation, the higher is the FDG intake [21]. The different expression pattern of glucose transporter 1 (GLUT1), hexokinase, and NIS are known to be related to the variable uptake of RAI and FDG [22, 23].

DTC has a wide spectrum of cancer cellular differentiation. The problem is that we cannot know the accurate differentiation state of thyroid cancer on the basis of only the RAI scan. In addition, dosimetry using I-124 PET/CT is necessary to calculate the amount of RAI concentrated in metastatic lesions, but it is not practical for clinical application. However, when FDG PET is taken along with RAI WBS, we can obtain information on the degree of the uptake of two radiotracers, from which we can infer the differentiation state of thyroid cancer. This process can also be used to predict treatment response to RAI.

Currently, a diagnosis of radioactive iodine-refractory (RAIR) DTC is made based on the clinical evidence that the patient does not respond after a cumulative ^131^I of 600 mCi (222,000 MBq) or more [6, 24]. That is, to be diagnosed as RAIR-DTC, the patient should receive at least 3 RAI therapies, and considering the 6-month treatment interval, it will take over two years to establish this diagnosis. This may lead to the patient being deprived of the opportunity to receive an appropriate treatment early. According to our research, FDG PET has a possibility to be useful as an early predictor. Early identification of RAIR-DTC with FDG PET/CT can make administration of alternate therapies such as surgical resection or TKIs to the patients in the proper time [24].

In lesion-based analysis with subgroup, RAI uptake was well correlated with DCR. In addition, uptake grades of both RAI and FDG were well correlated with DCR. From these results, we can see that it is important for a sufficient amounts of RAI to enter the cell to kill cancer cells. In addition, the correlation between FDG uptake grade and therapeutic effect well demonstrates the therapeutic effects according to the degree of cellular differentiation. In each lesion-based analysis, there was no significant parameter having correlation with RR.

FDG uptake was not significant negative predictor in the lesion-based analysis. The reason may be the difference in the evaluation method of treatment response. In the total patient group, the positive therapeutic response of RAI was defined as reduced lesion size or decrease of serum Tg levels. In the lesion-based analysis, however, serum Tg level could not be included for the therapeutic response evaluation because it represented overall response and not individually for each lesion.

When uptake patterns of RAI and FDG in each metastatic lesion were evaluated, 1/4 to 1/3 of patients showed inter-lesional heterogeneity between several metastatic lesions. That is, all metastasis may not be simultaneously treated by RAI therapy. These findings indicate that local control using surgery or external radiation of the metastatic lesions can be sometimes quite important in the treatment strategy of DTC. In many cases, the main causes of death in DTCs are local problems, such as malignant pleural effusion, pneumonitis and asphyxia by laryngeal nerve or tracheal invasion [25, 26]. Therefore, we would like to emphasize the lesionalized therapy beyond personalized therapy especially in DTC [26]. More researches are needed in regarding to this issues.

The limitation of this study is that it was retrospectively investigated. Also, a relatively small number of patients were enrolled in this study. Further validation with a large number of prospective cohort patients is required. Lastly, all metastatic lesions could not be pathologically confirmed due to clinical limitations. This is a problem that needs to be solved for all of us to study tumor metastasis.

In conclusion, post-therapy RAI scan alone was not sufficient to predict RAI therapy response in patients with metastatic DTC. The patients with FDG avid-metastasis showed poor response to RAI therapy regardless of the degree of RAI uptake. These findings imply that FDG PET/CT provides additional information in the prediction of RAI therapy response and further contributes to the establishment of a proper therapy strategy for metastatic DTC in early period.

## Supporting information

S1 File This is the dataset for this study. (XLSX)
